# Magnetic field effects on behaviour in *Drosophila*

**DOI:** 10.1038/s41586-024-07319-x

**Published:** 2024-05-01

**Authors:** Steven M. Reppert

**Affiliations:** Department of Neurobiology, UMass Chan Medical School, Worcester, MA USA

**Keywords:** Neuroscience, Sensory processing

arising from: M. Bassetto et al. *Nature* 10.1038/s41586-023-06397-7 (2023)

The fruit fly *Drosophila melanogaster* is a model organism that has been used by several laboratories to study geomagnetic sensing and its molecular basis. Bassetto et al.^1^ proclaim that there is no evidence for magnetic field effects on behaviour in *Drosophila*. I challenge their conclusion and defend the work in Gegear et al. 2008 (ref. ^2^), in which a binary-choice T-maze assay not only was used to reveal fruit fly magnetosensitivity but also provided mechanistic insights into the role of the ultraviolet-A/blue-light photoreceptor cryptochrome (Cry) in the magnetic response. Reviewing all of the published data, there is considerable evidence for magnetosensitivity in fruit flies.

Gegear et al. 2008 (ref. ^2^) developed a viable *Drosophila* behavioural assay for assessing magnetosensitivity at a field intensity of 500 µT. In an illuminated apparatus, flies experience a magnetic field generated by an electric coil system and exhibit their magnetosensitivity in a binary-choice T-maze^2–4^. The two-coil system is ideal for behavioural studies of magnetosensitivity, because it produces a magnetic field on one side of the T-maze, while producing no field on the opposite side. Importantly, the studies were carried out in the same laboratory where olfactory conditioning controls were routinely carried out in which flies are trained to associate odours with sugar reward. In the T-maze assay, wild-type flies showed significant naive and trained responses to the magnetic field, and the responses were light dependent. The ultraviolet-A/blue-light photoreceptor Cry^5^ mediated the light-dependent magnetosensitivity. In a second study, Gegear et al. 2010 (ref. ^3^) showed that when a *cry* transgene is properly expressed in Cry-deficient flies, a full magnetic response with appropriate light activation is restored. All of the data discussed herein are from published resources.

Any behavioural paradigm is sensitive to the environment in which it is carried out and this is particularly the case for fly conditioning. It is arguably the most complex of these types of *Drosophila* phenotype and requires considerable skill and experience to obtain reliable results. Although the experiments of Bassetto et al.^1^ might have been optimally shielded against interfering outside magnetic effects, it is evident in their Methods section that the critical ‘positive conditional control’ utilizing olfactory conditioning was not carried out under the same conditions as the failed magnetic conditioning studies. Instead, these ‘controls’ were carried out under temperature- and humidity-controlled conditions in Oxford, UK. Without ‘controls’ under the same location and conditions, it is impossible to determine whether the shielded location in Oldenburg, Germany, had the appropriate environment (humidity and temperature) that permits robust sugar-reinforced conditioning. The lack of an appropriate ‘positive conditional control’ in Oldenburg is a substantial criticism and suggests that there may be other important variables that differ between the studies in Bassetto et al.^1^ and those in Gegear et al. 2008 (ref. ^2^).

Bassetto et al.^1^ emphasize the large number of flies they tested (97,658) in the T-maze without finding a magnetic response, compared to the “small sample size” used in Gegear et al. 2008 (ref. ^2^). Notably, >39,500 flies were used to complete the studies in Gegear et al. 2008 (ref. ^2^). There were 390 groups of 100–150 flies used; the number of flies is easy to calculate from the data in the figures. This comparatively large number of flies used is in stark contrast to the small number of flies implied by Bassetto et al.^1^ and in the News and Views piece by Warrant^6^.

Bassetto et al.^1^ next reassessed the statistical analysis in Gegear et al. 2008 (ref. ^2^). Their reanalysis is off base and does not support the contention that most of the original results were not statistically significant and were instead false positives.

Bassetto et al.^1^ criticize the use of parametric statistical testing in the Gegear et al. 2008 paper^2^. However, analysis of *Drosophila* conditioning data is frequently carried out using parametric statistics. Indeed, Krashes and Waddell^7,8^ advise using parametric statistical testing of performance index values derived from appetitive and aversive olfactory conditioning assays and recommend a sample size of 8–10 replicates per condition per genotype. Instead, Bassetto et al.^1^ have selected an extremely conservative approach to reanalysis of the data in Gegear et al. 2008 (ref. ^2^). This choice leads to misguided conclusions on the statistical power of the original analysis.

When using an ordinal logistic fit model to assess the synthetic dataset, which is equivalent to the type of generalized linear model used by Bassetto et al.^1^ (based on the group averages in Gegear et al. 2008, Fig. 1b^2^; discussed in the text and Supplementary Fig. 1a of Bassetto et al.^1^), the statistical results are very dependent on how the batches of about 100 flies (in each experiment) are encoded in the model. With ‘batch’ included as an independent variable, the effect of training is minimal (*P* = 0.33), whereas omission of ‘batch’ altogether leads to a highly significant effect of training (*P* < 0.0001). Presumably, Bassetto et al.^1^ chose the former option.

Our conclusion that the approach of Bassetto et al.^1^ is overly conservative is based on a much more straightforward, non-parametric approach (the Wilcoxon rank sum test, also known as the Mann–Whitney *U*-test). The data for the naive and trained groups of flies in the synthetic dataset are highly significantly different by this analysis (*P* < 0.0001).

Mimicking the approach of Bassetto et al.^1^ to generate a single synthetic dataset, we generated an additional 20 synthetic datasets. When using the very conservative approach of Bassetto et al.^1^ (presumably a binominal approach with ‘batch’ as an independent variable), 5 of 20 datasets demonstrated a significant effect of training, whereas 15 did not. When using three other approaches (*t*-tests, ordinal logistic binominal models without ‘batch’ or non-parametric rank tests), all 20 synthetic replicates demonstrated highly significant differences between the groups (*P* < 0.0001). Thus, Bassetto et al.^1^ seem to have selected a statistical approach with extremely poor sensitivity for detecting differences when reanalysing the data in Gegear et al. 2008 (ref. ^2^). Their conclusion that the results in Gegear et al. 2008 (ref. ^2^) represent a ‘false positive’ is unfounded. Moreover, if false positives occurred in previous studies, they would be expected to occur in a variety of treatments and not in a way that consistently provides evidence for magnetosensitivity.

Bassetto et al.^1^ also criticize the statistical approach used in Gegear et al. 2008 (ref. ^2^) by stating that it assumes independence of each fly in a batch and subsequently treats each fly as an independent biological replicate, violating the requirement for independence of the samples and leading to pseudo replication. In fact, statistical analysis was carried out on the 8–12 independent values for performance index per group (each of which was derived from an independent batch of 100–150 flies). There is no pseudo replication.

Bassetto et al.^1^ were also unable to detect a magnetic effect on negative geotaxis in *Drosophila*, as reported in Fedele et al.^9^. Importantly, the magnetic response reported by Fedele et al.^9^ was replicated independently by Bae et al.^10^. This replication is not mentioned by Bassetto et al.^1^. Instead, they tried but were unable to replicate the work in Fedele et al.^9^. The inability of Bassetto et al.^1^ to replicate the work of not only Fedele et al.^9^ but also Bae et al.^10^ makes their negative results less convincing.

There are at least 15 papers over the past 50 years reporting the existence of a fly magnetic sense, and several of these suggest a Cry-based mechanism (papers listed in Bassetto et al.^1^). Most of these reports used assay systems other than the T-maze and negative geotaxis paradigms. Nevertheless, Bassetto et al.^1^ dismiss all of these other reports. Their refutation of these studies without direct evidence is unsubstantiated.

Bassetto et al.^1^ conclude by claiming that night-migratory songbirds (which are technically challenging for any kind of molecular genetic analyses) remain the organisms of choice for elucidating the mechanism of light-dependent magnetosensitivity. However, the authors overlooked the published work on the biologically relevant magnetic compass of the migratory monarch butterfly. Two independent reports that use distinctive behavioural assays show that individual monarchs manifest robust light-dependent inclination magnetic responses to Earth-strength magnetic fields^11,12^. Moreover, genetic studies show that the photoreceptive Cry1 protein is essential for the monarch’s light-sensitive magnetic compass^12^. The recent successful use of reverse genetics in monarchs^12^ indicates that the butterfly is an excellent choice for delineating the molecular mechanisms underlying light-dependent magnetosensing in the context of compass navigation.
